# Streamlining the Pipeline for Generation of Recombinant Affinity Reagents by Integrating the Affinity Maturation Step

**DOI:** 10.3390/ijms161023587

**Published:** 2015-09-30

**Authors:** Renhua Huang, Kevin T. Gorman, Chris R. Vinci, Elena Dobrovetsky, Susanne Gräslund, Brian K. Kay

**Affiliations:** 1 Department of Biological Sciences, University of Illinois at Chicago, 900 S. Ashland Ave., Chicago, IL 60607, USA; E-Mails: rhuang@meso-scale.com (R.H.); kgorma5@uic.edu (K.T.G.); guidotech@yahoo.com (C.R.V.); 2 Structural Genomics Consortium, University of Toronto, 101 College St., Toronto, ON M5G1L7, Canada; E-Mails: elena_dobrovetsky@yahoo.com (E.D.); Susanne.graslund@ki.se (S.G.)

**Keywords:** affinity maturation, affinity selection, error-prone PCR, FN3 monobody, loop shuffling, Kunkel mutagenesis, megaprimer, off-rate selection, phage-display, secondary library

## Abstract

Often when generating recombinant affinity reagents to a target, one singles out an individual binder, constructs a secondary library of variants, and affinity selects a tighter or more specific binder. To enhance the throughput of this general approach, we have developed a more integrated strategy where the “affinity maturation” step is part of the phage-display pipeline, rather than a follow-on process. In our new schema, we perform two rounds of affinity selection, followed by error-prone PCR on the pools of recovered clones, generation of secondary libraries, and three additional rounds of affinity selection, under conditions of off-rate competition. We demonstrate the utility of this approach by generating low nanomolar fibronectin type III (FN3) monobodies to five human proteins: ubiquitin-conjugating enzyme E2 R1 (CDC34), COP9 signalosome complex subunit 5 (COPS5), mitogen-activated protein kinase kinase 5 (MAP2K5), Splicing factor 3A subunit 1 (SF3A1) and ubiquitin carboxyl-terminal hydrolase 11 (USP11). The affinities of the resulting monobodies are typically in the single-digit nanomolar range. We demonstrate the utility of two binders by pulling down the targets from a spiked lysate of HeLa cells. This integrated approach should be applicable to directed evolution of any phage-displayed affinity reagent scaffold.

## 1. Introduction

There is a growing interest in generating recombinant affinity reagents as an alternative to immunization of animals for the purpose of basic research. Such reagents have a number of advantages [1]: they are renewable, they can be shared easily (*i.e.*, emailing the DNA sequences), they offer reproducibility in experiments [1,2], they can be genetically fused to other proteins or epitope tags, they are amenable to directed conjugation to small molecules or resins, and their affinity and specificity can be controlled. A variety of proteins have proven useful as scaffolds [3] for yielding affinity reagents, including affibodies [4,5], anticalins [6,7], avimers [8], designed ankyrin repeat proteins (DARPins) [9,10], fibronectin type III (FN3) monobodies [11,12,13,14], fragments of antigen binding (Fabs) [15,16,17], single-chain Fragments of variable regions (scFvs) [18,19], and single-domain antibodies [20,21]. Display technologies, such as mRNA display [22], phage display [23,24,25], ribosome display [26], and yeast display [27,28,29] are used to screen libraries by affinity selection, yielding clones that are specific and high-affinity for their cognate targets.

Often in a research effort, there is a desire to improve the affinity of a particular binder for its target. One effective strategy is to generate variants of the coding region of a single clone by error-prone PCR [30,31,32] or DNA shuffling [33,34,35,36], and construct a secondary library, from which one isolates tighter binding clone, by performing affinity selection with reduced amounts of target [37] and through off-rate selection [38,39]. This approach has been termed “affinity maturation”, as it mimics the result of somatic hypermutation, which occurs in immunoglobulin genes upon repeated exposure of animals to antigen [40].

As part of our ongoing efforts to streamline phage-display in the generation of recombinant affinity reagents, we decided to integrate affinity maturation into the pipeline by taking advantage of modifications that we have made to the Kunkel mutagenesis technique [41,42,43]. While this approach should be suitable for improving the affinity and/or specificity of recombinant affinity reagents based on various scaffolds, we apply it here to the FN3 monobody. This scaffold has 94 amino acids, and adopts a thermally stable three-dimensional structure composed of seven beta-sheets and three loops on two opposite sides of the domain [11]. From phage- and yeast-displayed libraries of the FN3 monobody, recombinant affinity reagents have been generated for a wide variety of targets, including Abl SH2 domain [44,45], Src SH3 domain [12], integrins [46], lysozyme [47], maltose binding protein [48], phosphorylated IkappaB alpha [49], and vascular endothelial growth factor receptor two [50]. Examples of this class of scaffold have already reached clinical trials [51]. In our hands, the DNA of a pool of phage-displayed FN3 monobody clones, which were affinity selected from the primary library, is used as template for error-prone PCR [30,42]. In this approach, the two parts of the FN3 monobody coding region, each encompassing the BC (The loop between B-strand and C-strand) and FG (The loop between F-strand and G-strand) loops, are amplified. The resulting DNA fragments act as “megaprimers” [42] to anneal to the single-stranded vector DNA. Once converted to double-stranded DNA, the sample can be electroporated into *Escherichia coli* (*E. coli*) bacteria, thereby generating secondary libraries with 10^8^ to 10^9^ diversity. Through three additional rounds of affinity selection, one can isolate variants with low dissociation constants (*K*_D_). In this manner, we can routinely generate high affinity (*i.e.*, *K*_D_ < 50 nM) monobodies to target proteins.

## 2. Results and Discussion

### 2.1. The Pipeline

**Figure 1:**
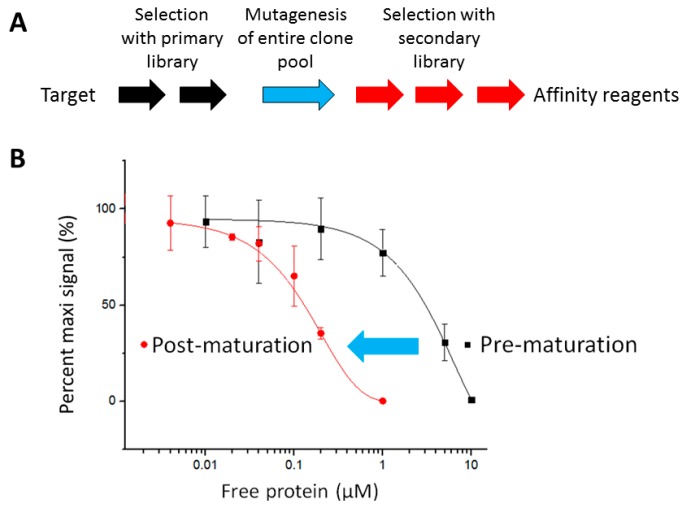
Integrating affinity maturation into the phage-display pipeline. (**A**) Schematic of the pipeline. After the target has been obtained, two rounds (Two black arrows) of phage-display affinity selection are performed, followed by creation of a secondary library via mutagenesis (A blue arrow). The resulting secondary library is then affinity selected for another three rounds (Three red arrows) to isolate individual clones that bind the target; (**B**) Competition polyclonal enzyme-linked immunosorbent assay (ELISA) illustrating the overall improvement in affinity of virion pool of post-maturation. The blue arrow indicates the mutagenesis process.

The growing demand for the recombinant affinity reagents in the scientific community is unmet due to the inefficient process to generate such reagents with superior affinities. To improve such process, we devised a method that integrated the affinity maturation step into the selection process (Figure 1A). In such a method, the entire output from affinity selection of the primary library is mutated, and the resulting secondary library is further selected for another three rounds. One of the impetuses of our efforts was based on emulating how affinity maturation is an integral part of mRNA or ribosome-display in generating affinity reagents. In either method, one can utilize error-prone PCR conditions for amplifying DNA from the pools of clones, followed by new rounds of affinity selection. This approach has led to the generation of DARPin binders with picomolar affinities to maltose binding protein [52], the ectodomains of Her2 [53], the tumor-associated antigen epithelial cell adhesion molecule, EpCAM [52], and the c-Jun N-terminal kinases [54]. From our efforts described here, one can now similarly accomplish the same with phage-display by taking advantage of the ability to use “megaprimers”, derived from PCR amplification of the coding region to prime DNA synthesis and generate heteroduplexed DNA for bacterial transformation and generation of a library of variants. Thus, our approach mimics aspects of recursive diversity, selection, and reproduction in Darwinian evolution.

With such a method, the affinity of the entire clone pool selected against mitogen-activated protein kinase kinase 5 (MAP2K5) was improved, as shown by the shift of the IC_50_ value (Figure 1B). The IC_50_ values of the virion pools of pre- and post-affinity maturation were approximately 3 μM and 150 nM, respectively. Thus, after maturation, the virion pool bound 20-fold tighter.

### 2.2. Affinity Selection and Mutagenesis

To test the general utility of this approach, we wanted to apply it to eleven human proteins (Table 1), which represent many different classes of proteins involved in cell signaling. All these proteins were well expressed in *E. coli*, obtained in pure form at the milligram level, and biotinylated. Our goal was to generate recombinant affinity reagents to the well-folded forms of the proteins and that have affinities (*i.e.*, *K*_D_ values < 50 nM) comparable to monoclonal antibodies.

**Table 1:** Eleven recombinant human antigens for affinity selection.

Targets	Full Names	Uniprot ID	Biological Processes	Antigens (Amino Acids #)
CDC34	ubiquitin-conjugating enzyme E2 R1	P49427	ubiquitin ligase activity	7–184
CDK2	cyclin-dependent kinase 2	P24941	cell-cycle control	3–286
COPS5	COP9 signalosome complex subunit 5	Q92905	deubiquitination, JNK signaling, secretion	9–309
CTBP1	c-terminal-binding protein 1	Q13363	corepressor of transcriptional regulators	20–440
MAP2K5	mitogen-activated protein kinase kinase 5	Q13163	scaffold for the formation of a signaling process	5–108
PAK1	p-21 protein activated kinase 1	Q13153	regulation of cell-proliferation, apoptosis	258–544
PLAA	phospholipase A-2-activating protein	Q9Y263	maintenance of ubiquitin levels	338–795
RAB6B	Ras-related protein-6B	Q9NRW1	retrograde membrane trafficking via Golgi	6–182
SF3A1	Splicing factor 3A subunit 1	Q15459	mRNA processing, mRNA splicing	423–790
TDP43	TAR DNA-binding protein 43	Q13148	regulation of transcription and splicing	1–106
USP11	Ubiquitin carboxyl-terminal hydrolase 11	P51784	deubiquitination, regulator of NF-kappa-B activation	61–285

**#** amino acid number.

**Figure 2:**
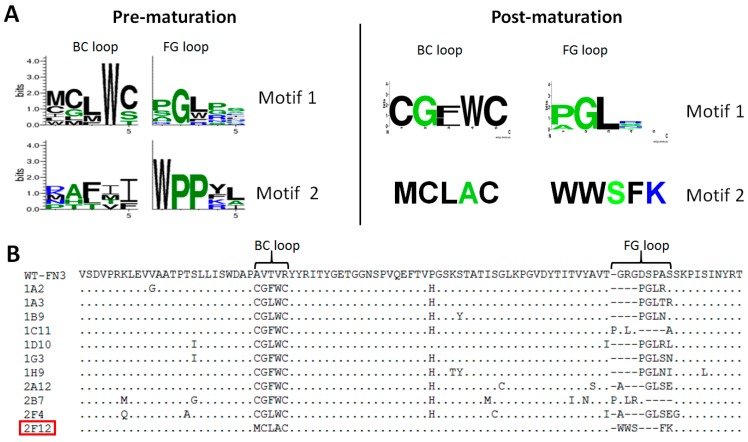
Binding motifs, loop sequences and framework mutations of binders to ubiquitin-conjugating enzyme E2 R1 (CDC34). (**A**) Sequence motifs shared among binders of pre- and post-affinity maturation. There are two motifs for binders of both pre- and post-affinity maturation. Two sibling clones were isolated for motif 2 of post-maturation. The motif logo plots were generated by WebLogo [55]. In the logo plot, the following amino acid residues are in green: A, G, H, P, S, T. Amino acid residues of R, K, and N are in blue and the rest of the residues are in black; (**B**) Sequence alignment of eleven binders of post-maturation. Clone 2F12 (Red-squared) is the only sequence of the motif 2 and the rest of clones belong to motif 1. The FG loop of wild-type FN3 (WT-FN3) has eight residues, which was shortened to five residues in FN3 variants. Dashes are inserted to maximize the sequence alignment.

Affinity selection of the primary library against the eleven recombinant proteins led to enrichment of FN3 monobodies that bound to their cognate targets, as shown by polyclonal ELISA after two rounds of selection (data not shown). To determine the enrichment level of the binders to each target, 94 individual clones were picked for phage ELISA. For each of the eleven targets, 2%–85% of their tested clones bound well to the targets in the phage ELISA, and sequencing of positive clones revealed that for some of the targets, there were 1–2 sequence motifs shared among their binders. Two such motifs are shown for binders to CDC34 (Figure 2A, Pre-maturation). Taken together, these results suggest that isolation of binding clones from the primary library to each target is successful and that the FN3 monobody is a valuable scaffold capable of generating affinity reagents against a wide variety of protein targets.

Out of the eleven targets, we selected six representative targets (*i.e.*, CDC34, COPS5, MAP2K5, PLAA, SF3A1, and USP11), which have various degrees of enrichment level of binders after primary library screening, to test our idea of integrating affinity maturation into the pipeline. We used a combined mutagenesis and loop-shuffling approach to construct secondary libraries. After transformation, the diversity of secondary libraries was calculated by multiplying the total number of transformants by recombination rate, which ranged from 10% to 32%. Due to the presence of the stop codons in the parental ssDNA template, the non-recombinant clones did not display FN3 monobody. The sizes of six secondary libraries ranged from 9 × 10^7^ to 1.5 × 10^9^. Sequencing of the mutant clones revealed that the sequences of BC and FG loops were shuffled and the framework regions contained point mutations (data not shown). The average mutation rate for the point mutations in the framework regions is 0.6% (6 point mutations per 1000 nucleotides).

One advantage of performing error-prone PCR across the entire coding region is that mutations will occur in the scaffold’s framework in addition to the randomized loops. It is well known that mutations in the framework can enhance the affinity of binders through several mechanisms [56]: introducing an additional site of interaction, altering the three-dimensional structure of regions or loops that make contact with the target, or increasing the thermal stability of the affinity reagent. We have observed the same result [57]: a single point mutation in the framework of one particular scFv accounts for a 300-fold improvement in affinity.

A second advantage of our method is that it is not limited by the potential bottleneck of using a single clone for affinity maturation. It is possible that the single clone chosen to be the basis for expansion and reselection is not the best possible binder and this may place a limitation on what can potentially be achieved with affinity maturation. On the other hand, with a pool of clones that have undergone two rounds of affinity selection, there will be a range of weak to strong binders, from which more diverse variants can be generated by error-prone PCR. Selection of such a variant pool will increase the likelihood of yielding tighter binders. With the latest development in deep sequencing [58] of DNA samples, we can envision in the future characterizing clone pools before and after mutagenesis and gain further evidence about how the diversity of the pools and the mutagenesis profiles influence the outcome of the affinity selection experiments.

### 2.3. Characterization of Affinity Matured Monobodies

**Figure 3:**
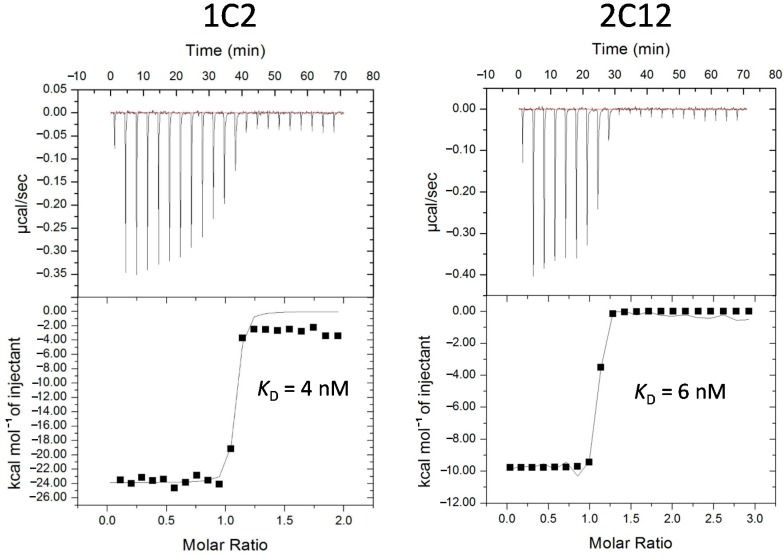
Determination of dissociation constant (*K*_D_) via isothermal titration calorimetry (ITC). Purified monobodies were injected into the sample cells loaded with their respective targets. Binding events elicited a heat change, which was then graphed as a function of molar ratio. GE Healthcare software (GE Healthcare; Piscataway, NJ, USA) was used to extrapolate the *K*_D_ value. Both clones exhibited affinities in the single-digit nanomolar range.

**Figure 4:**
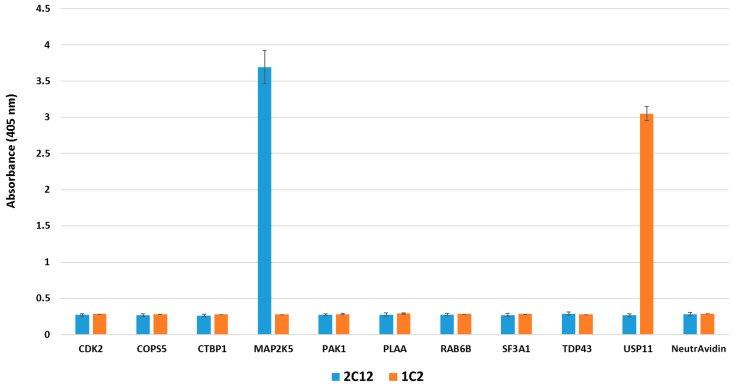
Affinity-matured monobodies bound specifically to their targets in enzyme-linked immunosorbent assay (ELISA). Virions displaying affinity-matured monobodies binding to mitogen-activated protein kinase kinase 5 (2C12) and ubiquitin carboxyl-terminal hydrolase 11 (1C2) were tested in phage ELISA for specificity against a panel of nine other biotinylated targets. NeutrAvidin was used to immobilize the targets to the well. Phage particles bound to the target were detected via anti-M13-horseradish peroxidase (HRP) antibody. Both clones specifically recognized their cognate targets.

The secondary libraries were affinity selected for three rounds, followed by a polyclonal ELISA to assess the enrichment of binders. All six resulting pools gave very strong signals in polyclonal ELISA (data not shown). For each target, 94 or 188 individual clones were also picked in phage ELISA to identify clones with the strongest signals in ELISA. Clones with the highest ELISA signals were selected for sequencing. For CDC34, sequencing revealed that its twelve matured clones had two common motifs (Figure 2A, Post-maturation) and they had many mutations in the framework (Figure 2B). While it is difficult to access the relative impact of the mutations in the framework with respect to target binding without careful follow-up experimentation, one particular framework mutation, Pro-51-His, was present among nine out of the twelve sequenced clones, suggesting that this shared mutation may have a strong positive contribution to enhanced affinity or stability. The best clones for CDC34, COPS5, MAP2K5, SF3A1 and USP11 were further characterized by competition ELISA, which showed that these monobodies had IC_50_ values ranging between 6 and 52 nM (Table 2). The coding regions of these monobodies were subcloned into a pET14B-SUMO fusion expression vector, and two monobodies, which were well expressed, 1C2 for USP11 and 2C12 for MAP2K5, were selected for affinity measurement using isothermal titration calorimetry (ITC) [59]. ITC analysis revealed that the *K*_D_ values for 1C2 and 2C12 were 4 and 6 nM, respectively (Figure 3). These affinity values are about 100-fold higher than those observed for the non-matured clones, which typically have values in the range of micromolar to high-nanomolar. These improved affinities match those of good antibodies [60] and put them among the tightest monobodies generated by phage display [44,48,61]. To evaluate the binding specificities of 1C2 and 2C12 to the targets, phage ELISA was performed, in which both monobodies bound specifically to their cognate targets (Figure 4).

**Table 2:** Output sequences and affinities of clones after affinity maturation.

Targets	Clones	BC Loop (26–30)	FG Loop (77–81)	Framework Mutations	Affinity (nM)	
					ELISA	ITC
CDC34	1D10	CGLWC	PGLRL	S17I, T76I	<50	N/D
COPS5	1D7	RRWDV	WGIII	None	<10	N/D
MAP2K5	1C4	CRKCL	RLEWL	P51H, K83N	6	11
	2C12	CRKCL	RLEFL	None	17	6
SF3A1	1E2	ALPVY	VWWYE	None	<50	N/D
USP11	1C2	WWVPQ	PGIYQ	L18M, G61C, G65D, S82I	N/D	4
	1A9	WWSVP	PGIYA	D67V, S82I, Y92C	52	N/D

BC and FG loops are the variable regions. Estimated affinities were determined by competition; phage ELISA and/or isothermal titration calorimetry (ITC). N/D: experiments were not; performed to determine the values.

Although binders with single-digit nanomolar affinities were generated by our method, one might ask how this protocol can be improved in the future. We have several ideas. First, as we failed to generate binders of high affinity for PLAA, which happened to have only 2% binders after screening the primary library, we think that the low enrichment level can be a potential reason for the failure to generate high affinity binders. Therefore, for more challenging targets like PLAA, it is desirable to perform additional round of selection to further enrich the binder before mutagenesis. Second, as observed in another study [47], mutations in framework may lead to higher affinity, but they may also reduce the scaffold’s thermostability, resulting in poor expression [62]. We have similar finding in this study and it suggests that additional selection pressure, such as heat treatment, can be applied to mutagenic pools to select clones that not only have higher affinities, but also remain thermostable. Third, the sizes of secondary libraries constructed in this study are limited by the low recombination rate with the Kunkel mutagenesis, and thus one might increase the frequency of recombinant clones in the secondary library by restriction enzyme digestion [63] or by rolling circle amplification [64].

### 2.4. Pull-down Experiments with a Spiked HeLa Cell Lysate

**Figure 5:**
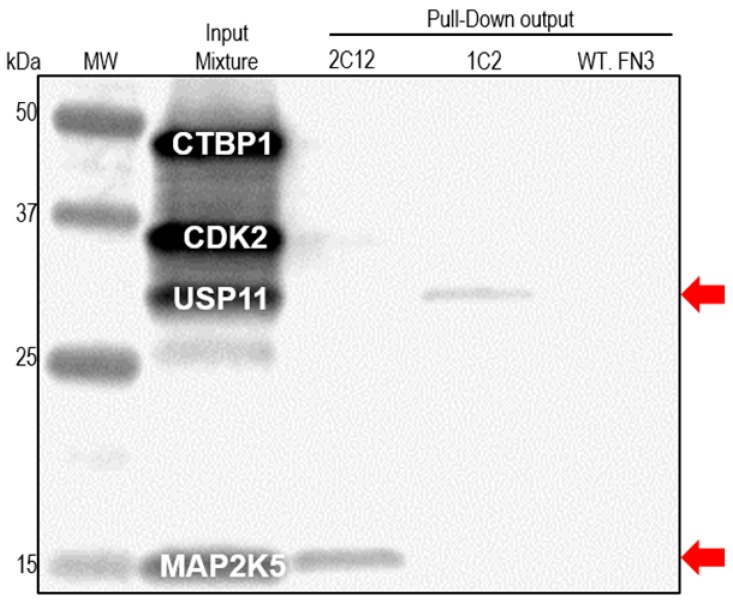
Pull-down of biotinylated targets from spiked HeLa cell lysate by affinity-matured monobodies. Four biotinylated targets were mixed (1 pmol each) with HeLa cell lysate (10 µg). FLAG-tagged FN3 monobodies were added to the mixture, and subsequent target/monobody complex was pulled down via anti-FLAG antibody-coated paramagnetic beads. After washing, the target/monobody complex was eluted off the beads and used in Western blot. Biotinylated targets were detected via a streptavidin-infrared dye conjugate. Red arrows indicate the positions of two pull-downed targets on the blot.

To demonstrate the utility of our reagents to recognize the target in complex mixture, we performed pull-down experiments. HeLa cell lysate was spiked with 1 pmol each of four biotinylated targets, and then incubated with 10 pmol of FLAG-tagged FN3 monobody. Target/monobody complex was later captured with anti-FLAG antibody-coated paramagnetic beads, resolved by SDS-PAGE, and transferred to a Polyvinylidene fluoride (PVDF) membrane for Western blot. By reacting the blot with a streptavidin-infrared dye, any biotinylated protein that was pulled down would be detected. As seen in Figure 5, the MAP2K5 binder, 2C12, was able to pull down 33% of the input protein, and the USP11 binder, 1C2, was able to pull down 7%. As these experiments were performed with only a 10-fold molar excess of the binder, increasing the binder-to-target ratio should improve the efficiency of target recovery. Nevertheless, the binders were able to recognize the targets in a very complex mixture, as well as not bind to the unrelated biotinylated targets.

## 3. Experimental Section

### 3.1. Subcloning, Overexpression and Purification of Antigens and Monobodies

For making the expression constructs of PAK1 and TDP43 for intracellular production in *E. coli*, their coding regions were amplified by PCR and subcloned into pET14-b (Novagen; Madison, WI, USA), which carries a hexahistidine tag for purification [65] and a small ubiquitin-like modifier (SUMO) tag for improved expression [66]. Plasmids with the full-length coding regions for PAK1 and TDP43 were obtained from Dr. Brian Kuhlman at the University of North Carolina at Chapel Hill and Dr. Raymond Roos at University of Chicago, respectively. The expression constructs of the other nine antigens were made in p28BIOHTEV-LIC with a hexahistidine tag for purification and an Avitag for *in vivo* biotinylation [67,68]. The coding sequences of the nine antigens were subcloned from cDNA (Mammalian Gene Collection, Toronto, ON, Canada) by PCR amplification, products of which were inserted into a BseRI linearized vector using the In-Fusion Cloning Kit (Clontech; Mountain View, CA, USA) and verified by DNA sequencing. The affinity matured FN3 monobodies were cloned to another pET14-b plasmid, which carries a FLAG-tag, a hexahistidine tag, and a SUMO tag.

The expression and purification of the proteins of PAK1, TDP43 and monobodies were described elsewhere [69]. The purified proteins of PAK1 and TDP43 were chemically biotinylated as described before [70]. The expression, purification and biotinylation of the other nine antigens were described in another study [71].

### 3.2. Affinity Selection of the Primary Library

The primary library has a diversity of 1.3 × 10^10^, which was constructed in a previous work [69]. For the affinity selection of the primary library, first, multiple centrifuge tubes were blocked overnight by casein (Thermo Fisher Scientific; Waltham, MA, USA) at 4 °C. The next day, streptavidin-coated paramagnetic beads (Promega; Madison, WI, USA) were washed three times with phosphate buffered saline (PBS; 137 mM NaCl, 3 mM KCl, 8 mM Na_2_HPO_4_, 1.5 mM KH_2_PO_4_), followed by addition of 1.5 nmol biotinylated target protein and tumbling for 30 min. The streptavidin-coated beads with the captured proteins were blocked with casein (Thermo Fisher Scientific) for 1 h, followed by blocking with 100 µM free biotin for 15 min and another three washes with PBS. Incubation of the phage library with the target took place in the blocked centrifuge tubes. After 2 h tumbling at room temperature, the streptavidin-coated paramagnetic beads were captured with a magnet and washed three times with PBS plus 0.1% Tween 20, and then another two washes with PBS. The steps of eluting bound phage virions, infecting TG1 cells (Lucigen; Middleton, WI, USA), collecting infected cells, and phage replication from the infected cells were performed as described previously [69].

The second round of affinity selection was conducted similarly as the first round selection, except with the following minor changes. The affinity selection was done by mixing the phage virions directly with the biotinylated proteins at a final concentration of 300 nM. After 1 h tumbling at room temperature, the blocked streptavidin-coated paramagnetic beads (Promega) were added to capture the protein-phage complex for 30 min on tumbler. Then the paramagnetic beads were collected with a magnet and washed three times with PBS plus 0.5% Tween 20, three times with PBS plus 0.1% Tween 20, and four times with PBS. The output from the second round selection was used for polyclonal phage enzyme-linked immunosorbent assay (ELISA) to determine if binders to the intended targets had been enriched. The details of the ELISA experiment can be found elsewhere [69].

### 3.3. Secondary Library Construction and Affinity Selection

Plasmid DNA was recovered from the virion-infected bacterial cells and used as the template for performing error-prone PCR, as described [42], with Mutazyme II DNA polymerase (Agilent; Santa Clara, CA, USA). For each target, two separate error-prone PCR reactions were performed to yield two DNA fragments. One fragment (145 bp) encompasses BC loop sequences and some flanking framework regions and the second fragment (161 bp) encompasses FG loop sequences and some flanking framework regions. The two pairs of primers for performing the error-prone PCR are as follows: the first pair (For amplifying BC loop fragment) includes forward primer, 5ʹ-acaagcttgctagcgccatgg-3ʹ and reverse primer, 5ʹ-ccaccggtttcaccgtacgtg-3ʹ; the second pair (For amplifying FG loop fragment) includes forward primer, 5ʹ-ccccggttcaggagttcactgtac-3ʹ and reverse primer, 5ʹ-gtcgacgcggccgct-3ʹ. The amplified double-stranded DNA fragments were purified with QIAquick PCR purification kit (Qiagen; Valencia, CA, USA) and used as “megaprimers” for generating covalently-closed circular DNA (cccDNA), which was used to electroporate TG1 cells (Lucigen), as described previously [42]. For each secondary library, the output of four to six electroporations were pooled and spread on 2 × YT (16 g/L tryptone, 10 g/L yeast extract, 5 g/L NaCl) agar plate containing carbenicillin (50 µg/mL) for overnight incubation at 30 °C. The next day the cells were scraped off the agar plate, inoculated into 2 × YT culture medium and infected with M13-KO7 helper virus for overnight phage amplification. The next day, the amplified phage virions were processed as described [42] for affinity selection.

The secondary libraries were subjected to another three rounds of affinity selection. The first round was conducted similarly as the selection of primary library. For the second round of selection, the phage virions were mixed with the biotinylated target at a final concentration of 30 nM and tumbled for 1 h, followed by addition of 6 µM non-biotinylated target for 1 h off-rate selection [39]. The complex of virion-biotinylated protein was captured by streptavidin paramagnetic beads (Promega), which were then washed 15 times with PBS supplemented with 0.1% tween 20 and 300 nM non-biotinylated target. The bound virions were processed for next round selection as described [69]. The third round of selection was performed similarly as the second round except that the concentration of the biotinylated protein was reduced to 10 nM for selection. For each target, the entire clone pool after third round selection was used for polyclonal ELISA. After polyclonal ELISA, single bacterial colonies were picked for phage ELISA to identify individual clones with the highest ELISA values.

### 3.4. Competition ELISA to Estimate Binding Strength

To estimate the half-maximal inhibitory concentration (IC_50_) of the phage-displayed monobodies for their target, a competition ELISA assay was performed for the clone pools selected against MAP2K5 before and after affinity maturation. Varying concentrations (5 nM to 10 µM) of non-biotinylated target protein was incubated for 1 h with virions, and then transferred to microtiter plate wells coated with biotinylated target. The ELISA signal of triplicate wells was measured and the average values of the readings were converted into percentage of maximum ELISA signal, at which there was no competition. The data were fitted with Origin9 (Originlab.com) software. For individual clones that had the highest ELISA values, a similar competition ELISA was performed to estimate their IC_50_ values.

### 3.5. Isothermal Titration Calorimetry (ITC)

FN3 monobodies and their respective targets were purified to homogeneity of >90% and dialyzed together against 25 mM Tris-HCl (pH 7.5), 150 mM NaCl and 100 mM imidazole. Their final concentrations were measured with a NanoDrop ND-1000 spectrophotometer (Thermo Fisher Scientific). Binding parameters were determined using a MicroCal ITC200 System (GE Healthcare). The FN3 monobodies were loaded into the syringe at 200 µM and their respective targets were loaded into the cell at 22 µM concentration. The reference well was loaded with water. FN3 monobodies were injected into the cell with a volume of 1.8 µL per injection at 25 °C, with a reference power of 10 mcal/s. The heat change of each injection was recorded, and analyzed with Origin software (GE Healthcare).

### 3.6. Pull-down Assays

A lysate of HeLa cell (10 µg; Active Motif; Carlsbad, CA, USA) was mixed with 1 pmol of four biotinylated proteins for 30 min at 4 °C. Then 10 pmol of purified FLAG-tagged FN3 protein was added to the target/lysate mixture, and tumbled for another 2 h at 4 °C. Meanwhile, anti-FLAG antibody coated magnetic beads (Sigma-Aldrich; St. Louis, MO, USA) were washed three times with PBS, blocked with casein (Thermo Fisher Scientific) for 1 h, and added to the mixture for capturing the complex of target/monobody. After that, beads were washed with RIPA buffer (25 mM Tris-HCl of pH 7.5, 150 mM NaCl, 1% NP-40, 1% sodium deoxycholate, and 0.1% SDS) in Kingfisher magnetic bead processor (Thermo Fisher Scientific). The remaining bound complex of target/monobody was eluted by heating at 95 °C for 5 min, resolved on Criterion TGX Stain-Free gel (Bio-Rad; Hercules, CA, USA), and later transferred to Polyvinylidene fluoride (PVDF) membranes. The membranes were probed with a streptavidin-IR dye conjugate (Rockland; Limerick, PA, USA) and scanned using an Odyssey FC imager (LI-COR Biosciences; Lincoln, NE, USA). The percent recovery was determined using ImageJ software (Bethesda, MD, USA) [72].

## 4. Conclusions

In this study, through affinity selection of a primary phage-display library, we are able to isolate FN3 monobodies that bind 11 distinct antigens. For five antigens, with a “megaprimer” method of integrating affinity maturation into the affinity selection, we successfully generate monobodies with low nanomolar affinities that rival those of high-quality antibodies. This affinity-maturation method uses binder pools for mutagenesis, which inserts point mutations and shuffles binding loops in the mutants simultaneously. Two monobodies, 1C2 and 2C12, bind specifically to USP11 and MAP2K5, respectively, in ELISA and pull down their targets from spiked lysates, making them potentially useful tools for studying their cognate targets in biological context. This method can also be used to engineer similar scaffold proteins, as well as antibody fragments, for affinity reagents that have wide applications in life science research.
